# Historical and current perspectives on blood endothelial cell heterogeneity in the brain

**DOI:** 10.1007/s00018-022-04403-1

**Published:** 2022-06-20

**Authors:** Ryota L. Matsuoka, Luke D. Buck, Keerti P. Vajrala, Rachael E. Quick, Olivia A. Card

**Affiliations:** 1 Department of Neurosciences, Lerner Research Institute, Cleveland Clinic, Cleveland, OH 44195 USA; 2 Department of Molecular Medicine, Cleveland Clinic Lerner College of Medicine, Case Western Reserve University, Cleveland, OH 44195 USA; 3 Present Address: Kansas City University College of Osteopathic Medicine, Kansas City, MO 64106 USA

**Keywords:** Brain vascularization, Angiogenesis, Cell diversity, Blood–brain barrier, Fenestrations, Neurological diseases, Vascular therapy

## Abstract

Dynamic brain activity requires timely communications between the brain parenchyma and circulating blood. Brain–blood communication is facilitated by intricate networks of brain vasculature, which display striking heterogeneity in structure and function. This vascular cell heterogeneity in the brain is fundamental to mediating diverse brain functions and has long been recognized. However, the molecular basis of this biological phenomenon has only recently begun to be elucidated. Over the past century, various animal species and in vitro systems have contributed to the accumulation of our fundamental and phylogenetic knowledge about brain vasculature, collectively advancing this research field. Historically, dye tracer and microscopic observations have provided valuable insights into the anatomical and functional properties of vasculature across the brain, and these techniques remain an important approach. Additionally, recent advances in molecular genetics and omics technologies have revealed significant molecular heterogeneity within brain endothelial and perivascular cell types. The combination of these conventional and modern approaches has enabled us to identify phenotypic differences between healthy and abnormal conditions at the single-cell level. Accordingly, our understanding of brain vascular cell states during physiological, pathological, and aging processes has rapidly expanded. In this review, we summarize major historical advances and current knowledge on blood endothelial cell heterogeneity in the brain, and discuss important unsolved questions in the field.

## Introduction

Throughout the body, blood vessels deliver oxygen and nutrients while removing metabolic wastes. In the brain, continuous and coordinated blood supply and waste clearance ensure the constant engagement of brain cells in processing enormous amounts of environmental stimuli and executing commands. To meet the high energy demands of the brain, an elaborate network of blood vessels is formed during development and maintained afterwards. Brain vasculature is organized in a specialized manner to support the diverse functions of the brain while protecting it from harmful blood-borne factors [1, 2]. Similar to peripheral blood vessels, vascular endothelial cells (vECs), the innermost layer of blood vessels, are covered by perivascular cells, such as smooth muscle cells and pericytes, to build functional brain vasculature. A notable difference in the brain as compared to other tissues is that vascular cells form close associations with neurons and glial cells to develop the neurovascular unit (NVU)—a brain–vascular interface where the blood–brain barrier (BBB) forms to limit cellular and molecular transport into the brain parenchyma [3, 4]. Most blood vessels in the brain establish this semi-permeable barrier, while others lack BBB properties and instead develop more permeable (fenestrated) phenotypes [5, 6]. Regardless of this heterogeneity in barrier properties, blood vECs in the brain are broadly classified into three types based on structural, molecular, and functional features: arterial, venous, and capillary endothelium [7–9]. Arterial and venous ECs share multiple conserved markers between the brain and other organs, while more prominent transcriptional heterogeneity is noted for capillary ECs [10]. A greater extent of organotypic features at the level of capillaries along the arterial–capillary–venous axis raises the question of how endothelial phenotypes are uniquely specified along this axis to meet organ-specific needs. However, the mechanisms by which brain vECs are specified into distinct subtypes within an interconnected brain vascular network have been understudied, and therefore remain unclear.

Across species, brain barriers are critical interfaces between the brain parenchyma and circulating blood [11–15]. Thus, disruptions in barrier properties of brain vasculature can be detrimental to brain health. For instance, current evidence has illuminated age-related changes in brain vascular structure and function. Declines in capillary density, reduced angiogenic potentials, decreased blood flow, impaired barrier properties, and vascular hypoperfusion are all hallmarks of aging brains [16–18]. These age-induced vascular declines, along with increased BBB permeability, can trigger neuroinflammation, which may lead to neurodegeneration and subsequent neurological deficits [19, 20]. A recent study suggests that counteracting the insufficiency of vascular endothelial growth factor (VEGF) signaling that occurs as animals age may prevent these vascular declines, thereby promoting healthy aging and extended life spans [21]. Other studies have proposed that age-induced shifts in endothelial transcellular transport machinery [22] and increased senescence in brain vECs [23] are potential underlying mechanisms of the endothelial dysfunction that causes BBB breakdown [22, 23]. However, it remains unknown whether aging has a specific or global impact on distinct subtypes of brain vECs, and which structural and functional changes individual vEC subtypes experience with aging.

BBB breakdown is not only observed during brain aging, but it has also been documented during the progression of numerous neurological diseases, including Alzheimer’s disease (AD) and multiple sclerosis (MS) [24, 25]. Hence, therapeutic interventions which could mitigate BBB dysfunction have been pursued as promising treatment options to prevent the progression of these disorders [26–28]. For example, a recent study demonstrates that genetically engineered Wnt7 ligands, which are crucial for BBB integrity, can prevent BBB breakdown and disease progression in several neurological disease models, illuminating their pharmaceutical potential to protect BBB function in both aged and neurological disease states [26]. However, in light of studies showing that forced activation of canonical Wnt/β-catenin signaling in fenestrated brain vECs can partially convert them into a BBB state [29, 30], it remains unclear how BBB restoration therapies may affect physiological function of fenestrated brain vECs. A deeper understanding of brain vEC subtype dysfunction and restoration will help develop vEC type-specific vascular therapies for neurological disorders.

The purpose of this review is to provide an overview of the major advances in the field of brain vascular biology, thereby clarifying our current knowledge and setting the ground for future research. This review focuses on covering topics related to the heterogeneous nature of brain blood vECs and does not describe in great detail topics that have been thoroughly reviewed elsewhere, including BBB cell biology, physiology, and pathology [6, 25, 31–35].

## Heterogeneous permeability, anatomy, and transcriptional profiling of brain endothelial cells: from historical observations to current perspectives

The first evidence of brain barrier properties came from dye injection studies in embryos [36–39]. Vital dyes such as trypan red, Evans blue, methylene blue, and trypan blue were injected into embryos of different species, including rodents, rabbits, dogs, cats, guinea pigs, and chicks [36, 37, 39, 40]. These dyes stained almost all tissues throughout the body, but they were excluded from the cerebrospinal fluid and most parts of the central nervous system (CNS). Later studies discovered that the lack of staining in the brain was due to the barrier properties of brain vECs, marking the identification of the endothelial BBB [41–43]. Conversely, there are small regions of the brain localized in close proximity to the midline brain ventricular systems that were stained after vital dye injections [38, 39, 44–46]. These stained brain regions were the choroid plexuses (CPs) and circumventricular organs (CVOs), named by Helmut Hofer in 1958 [47].

The CVOs include the subcommissural organ (SCO), organum vasculosum of the lamina terminalis (OVLT), subfornical organ (SFO), median eminence (ME), area postrema (AP), pineal gland (PG), and neurohypophysis (NH) [5, 48]. All CVOs contain neural tissues responsible for neuroendocrine function. In contrast, the CPs do not contain a neural tissue, thus they are not typically classified as CVOs. In both the CPs and CVOs, capillary networks lack the BBB and tight junctions that create a paracellular barrier in vECs, permitting high vascular permeability. These structural and functional features are in stark contrast to the limited permeability of those that form the BBB in the rest of the brain. The exception to this high vascular permeability is the SCO which does not form fenestrated capillaries [49]. Previous studies noted vascular permeability differences between the sensory and secretory CVOs in the adult mouse brain [50, 51]. In these studies, the authors used tracers of various molecular masses and observed different degrees of extravascular leakage and diffusion of these tracers across and within the CVOs, indicating that vasculature in each CVO displays unique barrier properties [50, 51]. Tight junction protein ZO-1 immunolabeling displays discrete protein localization patterns associated with vECs across the CVOs, providing additional evidence of heterogeneous vEC barrier properties in the CVOs [52].

Ultrastructural analyses using electron microscopy have been the gold standard to date for characterization of vEC anatomical structures. Electron microscopic studies in the 1960s revealed the presence of specialized tight junctions in vECs of the cerebral cortex that form the BBB [41, 42, 53]. In the late 1900s, studies identified the presence of endothelial fenestrae in the NH [54, 55], the AP [56, 57], the PG [58, 59], the SFO [60, 61], the OVLT [62, 63], the ME [54, 64, 65], and the CPs [66, 67]. Additionally, electron microscopic studies in some of these CVOs suggest that the number of endothelial fenestrae increases during development following vessel formation [54, 55, 65, 67], indicating that the induction of endothelial fenestrations takes place progressively after vessel formation.

**Fig. 1:**
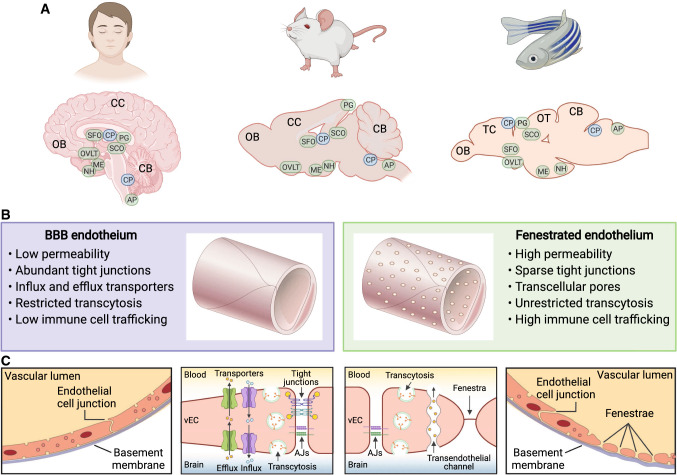
Comparative anatomy and function of BBB and fenestrated vECs between human, mouse, and zebrafish. **A** Schematic diagrams of the midline sagittal views of the brain in human, mouse, and zebrafish, indicating the locations of the CPs (blue) and CVOs (green). **B** Representative anatomical and functional features of BBB and fenestrated endothelium. **C** Schematic diagrams of the unique anatomical and functional features of BBB (left two panels) and fenestrated (right two panels) endothelium listed in **B**. *AJs* adherens junctions, *AP* area postrema, *BBB* blood–brain barrier, *CB* cerebellum, *CC* cerebral cortex, *CP* choroid plexus, *CVOs* circumventricular organs, *ME* median eminence, *NH* neurohypophysis, *OB* olfactory bulb, *OT* optic tectum, *OVLT* organum vasculosum of the lamina terminalis, *PG* pineal gland, *SCO* subcommissural organ, *SFO* subfornical organ, *TC* telencephalon, *vECs* vascular endothelial cells

The evolution of DNA sequencing and genome editing technologies since the late 1900s has facilitated molecular genetic studies in a variety of model organisms. Besides mammals, zebrafish are an excellent example of an emerging model organism that exhibits barrier and non-barrier properties across the brain, similar to mammals [13, 68, 69]. Moreover, the well-conserved anatomical features of resident cell types and endothelial fenestrations in the CVOs and CPs have been documented between mammals and zebrafish [48, 70–73]. The fruit fly, *Drosophila melanogaster*, has also recently emerged as a genetic model to study BBB biology [12, 74], although it is unclear whether or not this organism possesses fenestrated brain vECs. The use of a range of model organisms has compensated for the limitations of each individual organism, and allows insights into evolutionarily conserved mechanisms of brain barrier operations. Comparative anatomy and function of BBB and fenestrated vECs between the human, mouse, and zebrafish brain are illustrated in Fig. 1.

**Fig. 2:**
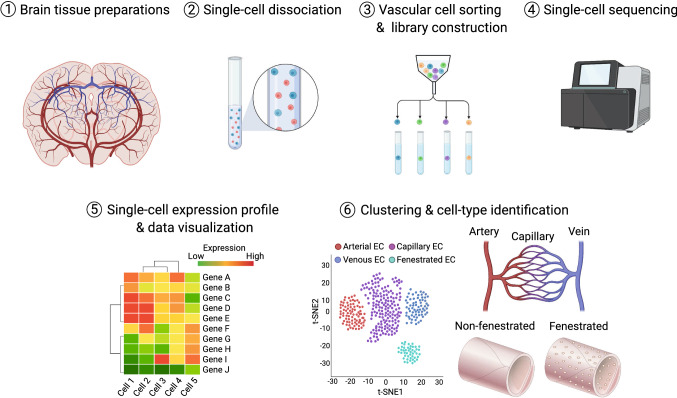
Flowchart summary of scRNA-seq analysis steps of isolated single vascular cells. This schematic flowchart provides a snapshot of the experimental workflow for scRNA-seq analysis. *ECs* endothelial cells

Most recently, cutting-edge next-generation sequencing platforms have offered genome-wide gene expression profiling at a wide range of sample scales and cellular resolutions. For instance, bulk transcriptomic analyses of vECs isolated from different organs using microarray and RNA-seq technologies have identified organ-specific, endothelial transcriptional signatures, including those unique to the brain [75–79]. Furthermore, single-cell RNA sequencing (scRNA-seq) of adult mouse and human brain vECs revealed gradual transcriptional changes along the artery–capillary–vein axis, a phenomenon known as zonation (Fig. 2) [9, 10, 80–82]. Even greater transcriptional differences were noted between BBB and fenestrated vECs [10]. Unsupervised clustering of the scRNA-seq data obtained from male and female adult mouse brain vECs revealed sex differences in brain endothelial cell transcriptomes [83, 84], indicating that sex is a crucial factor influencing transcriptional heterogeneity in brain vECs. These technologies have begun to be applied to the generation of brain vEC transcriptomes in many different contexts, which are discussed in later sections.

## Embryonic origins of brain endothelial cells

The initial steps of vascular development take place during gastrulation as mesodermal progenitors commit to an endothelial fate [85, 86]. vECs have been shown to derive from both the lateral plate mesoderm and the paraxial mesoderm (also known as the presomitic mesoderm) in zebrafish [87–89], chicks [90–92], and mice [93–95]. It was long believed that after the de novo formation of a primitive vascular network, new blood vessels arise exclusively by sprouting from pre-existing endothelial cells via angiogenesis [96, 97]. However, a recent study identified yolk sac-derived erythro-myeloid progenitors (EMPs) as another source of brain vECs in mice [98], challenging the current dogma that embryonic vessels expand solely by the proliferation of pre-existing endothelial cells. These findings have become controversial after a separate study found no evidence for the contribution of EMPs to brain vasculature using similar murine genetic tools [99]. It is presently unclear whether ECs derived from different embryonic origins are functionally distinct, or preferentially contribute to different brain vascular beds.

Previous studies in zebrafish indicated that vECs in the head derive from the anterior lateral plate mesoderm (ALPM) while those in the trunk and tail originate from the posterior lateral plate mesoderm [100–102]. A recent study employing retrospective cell-lineage tracing via light-sheet fluorescence microscopy implied that the dorsal–anterior side of the gastrula, early embryonic cell populations present even before the ALPM forms, is the major origin of vECs in the zebrafish head [102].

Despite these advances, how distinct brain vEC subtypes are specified and how they develop lineages from embryonic origins remain unanswered. Single-cell resolution fate mapping of whole brain vECs using a combination of lineage tracing, clonal analysis, time-lapse cell tracking, and omics approaches will help reveal vEC lineage trajectories from embryonic origins, filling this knowledge gap.

## Molecular and structural signatures of brain endothelial cell types

The BBB represents an evolutionarily conserved, highly selective separation at the interface between the circulatory system and brain parenchyma. To limit paracellular and transcellular molecular transport, BBB vECs establish a unique physical barrier characterized by the presence of tight junctions, an extensive transport machinery, and restricted transcytosis [25, 33, 34]. Additionally, the expression of leukocyte adhesion molecules in BBB vECs is maintained at low levels to limit the entry of immune cells into the brain parenchyma [25, 34]. Various isoforms of tight junction proteins belonging to the claudin family are expressed in brain vECs [103]; however, Claudin-5 is the most abundant claudin [103, 104], and its localization at BBB endothelial membranes and cell junctions is conserved across vertebrates [103, 105–107]. Conversely, Claudin-5 expression is undetectable in fenestrated brain vECs [29, 30, 73, 106, 108]. Thus, Claudin-5 has been used as a specific marker for BBB vECs. Other well-established specific markers for BBB vECs include the glucose transporter GLUT1, the docosahexaenoic acid transporter MFSD2A, the transcription factor LEF1, the tight junction proteins Occludin and ZO1, and the multi-drug resistance pump MDR1 [29]. The BBB vEC-specific expression of these genes relative to fenestrated brain vECs is also supported by recent transcriptomic data [29].

Plasmalemma vesicle-associated protein (PLVAP) is an endothelial cell-specific protein, and the only known molecular component of fenestral diaphragms and stomatal diaphragms of caveolae and trans-endothelial channels [109–111]. Fenestrae and trans-endothelial channels are transcellular pores that allow rapid exchange of molecules [111], while caveolae are spherical invaginations of the plasma membrane that play a role in transcytosis, the vesicular transcellular transport of macromolecules [112, 113]. Once vasculature becomes mature, PLVAP expression is restricted to fenestrated vECs in the brain, making it a unique molecular signature for these cells [10, 29, 72, 73]. PLVAP has also been implicated in controlling angiogenesis and immune cell trafficking in addition to its functions as a regulator of trans-endothelial molecular transport and vascular permeability via the formation of fenestral and stomatal diaphragms [72, 110, 111]. Inactivation and forced activation of endothelial-specific β-catenin in mice suggest a model whereby active β-catenin signaling suppresses PLVAP expression in brain vECs to limit their permeability, thereby establishing the BBB [29, 30, 114].

Over the past decades, studies have revealed that eliminating the unique molecular component(s) underlying BBB or fenestrated vEC properties leads to impaired vascular development and/or integrity. For example, Claudin-5 deficiency in mice resulted in vascular leakage of small molecular tracers in the brain, while endothelial tight junctions at the BBB were largely preserved [115]. Additionally, recent studies showed that haploinsufficiency or endothelial-specific deletion of *GLUT1* in mice leads to impaired angiogenesis and diminished vascular complexity in the thalamus [116] and the cerebral cortex [117], in addition to reduced pericyte coverage resulting in increased BBB leakage [118, 119]. Lastly, in mice and zebrafish deficient for PLVAP or its zebrafish ortholog, impaired formation of fenestral and stomatal diaphragms led to excessive or accelerated transcellular transport of blood-borne proteins through fenestrated vasculature [72, 120, 121].

## Cellular and molecular control of brain endothelial acquisition of unique properties

Over the last two decades, substantial progress has been made with regards to our understanding of the molecular mechanisms governing brain vascularization and endothelial cells’ acquisition of BBB and fenestrated properties. Brain vascularization is initiated by angiogenic sprouting from the peri-neural vascular plexus (PNVP), a primitive vascular network that covers the entire surface of the neural tube, into the brain parenchyma [122–124]. Paracrine VEGF-A signaling derived from the developing CNS is critical for PNVP formation and subsequent blood vessel invasion, branching, and density in neural tubes across multiple vertebrate species [125–130]. Genetic loss of heparin-binding VEGF-A isoforms (*VEGF*^*120/120*^ mice that are genetically engineered to produce solely the soluble isoform VEGF-A_120_) led to a significant reduction in vessel branch points and complexity in the brain [131], demonstrating VEGF isoform-specific control of brain vascularization.

Classical chick-quail transplantation experiments suggested that BBB barrier properties are not intrinsic to brain vECs, but rather, they are induced and maintained by neural environmental signals [132, 133]. These studies showed that neural tissues transplanted into the coelomic cavities are sufficient to induce BBB characteristics in mesenteric vessels, whereas brain vessels do not display BBB properties when invading into somite tissues transplanted into the brain ventricles [132, 133]. Isolated brain vECs in culture exhibit a rapid loss of BBB-specific transcripts and chromatin features [134], supporting the notion that neural environmental signals are necessary for vECs to maintain BBB properties.

## Development and maintenance of endothelial BBB properties

*Wnt/β-catenin signaling* Canonical Wnt/β-catenin signaling has been well established as a central regulator of brain angiogenesis, as well as of the induction and maintenance of endothelial BBB properties. Transgenic Wnt/β-catenin reporter mice demonstrated the specific activation of Wnt/β-catenin signaling in CNS vECs [114, 135, 136], but not in those of other organs [135]. Recent work revealed that β-catenin activities in fenestrated vECs of the CPs and CVOs are maintained at much lower levels than in those forming the BBB in both mouse and zebrafish models [29, 30]. Mechanistically, endothelial cell-specific deletion of *β-catenin* in developing mice results in a drastic reduction of BBB-specific proteins, including Claudin-5 and GLUT1, while leading to increased expression of the fenestrated vEC marker PLVAP [114, 135, 136]. Conversely, endothelial cell-specific β-catenin stabilization leads to the opposite outcomes in these gene/protein expressions [114]. Moreover, β-catenin stabilization in fenestrated vECs of the CPs and CVOs is sufficient to partially convert them into BBB-like states in vivo [29, 30], although it is unable to restore the loss of the BBB transcriptional and chromatin landscapes in primary brain vECs after short-term in vitro culture [134]. The critical role of β-catenin in BBB maintenance was further demonstrated via its deletion in endothelial cells of postnatal or adult mice [137, 138]. Endothelial β-catenin signaling was also shown to regulate vascular pericyte coverage in the mouse brain via modulation of *Pdgfb* expression [139]. Altogether, these findings support a model in which endothelial β-catenin signaling is central to the induction and maintenance of BBB properties in vECs and inhibits the expression of the fenestration marker PLVAP to limit vascular permeability.

Two classes of β-catenin activators (Wnt7a/Wnt7b and Norrin) have been well characterized in mice, which redundantly direct brain angiogenesis and BBB formation/maintenance in a brain region-specific manner. *Wnt7a* and *Wnt7b* are expressed largely in overlapping domains, and the combined, but not individual, loss of these two genes results in severe brain angiogenesis defects in mice [135, 136]. Wnt7a overexpression in the neural tube in vivo, or in primary cultures of mouse brain endothelial cells in vitro, is sufficient to enhance GLUT-1 expression in brain endothelial cells [135, 136]. Functional redundancy between Wnt7a and Norrin in BBB maintenance was identified in the cerebellum where these double mutant mice displayed increased BBB permeability and elevated PLVAP expression in vECs [140]. The key components of Wnt7s/β-catenin signaling are the receptor complexes consisting of Frizzled, Lrps, Gpr124, and Reck [138, 141–144], while Norrin/β-catenin signaling requires Frizzled-4 [145, 146] and another co-receptor component called Tspan12 [140, 147]. A recent study has reported that the guidance cue Netrin-1 signaling through its Unc5B receptor is critical for activating endothelial β-catenin pathways to maintain BBB integrity in mice [148].

Similarly, the crucial roles of Wnt7/β-catenin signaling through Gpr124/Reck receptors in regulating brain angiogenesis and BBB formation have been well documented in zebrafish [26, 149–151]. Additionally, a study in zebrafish showed that brain angiogenesis and transcriptional induction of endothelial BBB differentiation occur simultaneously during development [152]. Whether brain vECs establish BBB or fenestrated functional properties in a simultaneous or progressive manner in relation to vessel formation remains an important question.

*Retinoic acid (RA) signaling* RA signaling is indicated to be another important inducer of BBB properties in brain vECs, although its role in vivo is not entirely clear. High concentrations of RA treatments in vitro can induce BBB properties in cultured murine brain endothelial cells [153]. Pharmacological inhibition of RA receptor signaling in pregnant mice at the stage of brain angiogenesis and BBB formation (E10.5–16.5) leads to increased vascular leakage of tracers in the brain, in addition to resulting in significantly reduced expression of several BBB marker genes [154]. Endothelial RA signaling acts upstream of the Wnt/β-catenin pathway and suppress β-catenin expression in vECs via transcriptional suppression and phosphorylation-dependent protein degradation [139]. In mice globally lacking the RA-biosynthetic enzyme Rdh10, reduced levels of RA production occur. Subsequently, Wnt signaling activation and Wnt-responsive gene expression were significantly diminished, while gene expression of endogenous Wnt inhibitors (*Dkk1* and *Sfrps*) was upregulated [155]. In zebrafish, pharmacological inhibition of the RA degrading enzymes, cytochrome P450 family 26 (Cyp26), led to increased expression of Claudin-5 in fenestrated hypophyseal vessels [71], indicating that increased levels of RA can induce BBB properties in these fenestrated vessels and that Cyp26-mediated RA degradation represses the induction of the BBB properties. Further investigations into the signaling crosstalk between RA and Wnt/β-catenin pathways in vivo will clarify the epistatic and redundant relationships between these two pathways in BBB function.

*Suppressed transcytosis* Brain vECs with BBB properties display low rates of transcytosis, the transcellular vesicular transport of macromolecules from one side of vECs to the other [156, 157]. Recent studies have revealed molecular components that actively suppress transcytosis specifically in brain vECs. Selective expression of the lipid transporter Mfsd2a was identified in BBB vECs [158, 159], and its genetic deletion results in impaired BBB function in mice [158] and zebrafish [160]. This BBB endothelial-specific expression of Mfsd2a is important to establish a unique lipid environment that inhibits caveolae-mediated transcytosis in these ECs, thereby maintaining BBB integrity [161]. Endothelial cell-specific deletion of *β-catenin* in mice results in significant reduction of Mfsd2a expression in the brain [162], while EC-specific β-catenin stabilization leads to the upregulation of Mfsd2a in fenestrated vECs of the brain [29]. A recent study has identified the extracellular-matrix protein Vitronectin secreted from brain pericytes as a crucial ligand that restricts endothelial transcytosis in a cell non-autonomous manner [163], illuminating a cellular mechanism by which transcytosis in BBB vECs is suppressed.

*Pericytes* Pericytes are an important cell type that induces and maintains endothelial BBB properties [164–166]. A recent scRNA-seq study investigated endothelial cell transcriptional changes in response to pericyte deficiency (70–80% brain pericyte loss) in adult brains of *Pdgfb*^*ret/ret*^ mice [167]. This study showed that pericyte deficiency led to a significant reduction of BBB endothelial gene expression, notably transporters such as *Mfsd2a*, while inducing the upregulation of *Plvap* and leukocyte adhesion molecule expression, resulting in BBB disruptions. Expression of many BBB markers (e.g., Claudin-5 and Glut1) remained unchanged in this pericyte deficiency model, indicating a specific role for pericytes in regulating BBB function. These gene expression changes in the adult brains are consistent with the earlier study that identified the critical role of pericytes in BBB induction during embryogenesis and described similar gene expression changes in embryonic brains of *Pdgfrb* mutant mice deficient for pericytes [164]. In this pericyte-deficient mouse model, 2 distinct modes of BBB disruptions were observed: widespread increase in vesicular transcytosis across brain vECs [166, 167] and focal disruption of tight junctions causing hotspot leakage [167]. Reduced endothelial expression of Angiopoietin 2 triggers the latter mode of BBB disruptions in the absence of brain pericytes [167].

Studies among several taxa revealed multiple embryonic origins of brain pericytes [168], including avians [169, 170], mice [171–173], and zebrafish [174]. A recent study suggests the existence of differences in morphology and distribution pattern of pericytes along capillary vessels across brain regions [175], indicating that pericytes from different developmental origins exhibit morphological and functional differences in regulating specific types and/or function of brain vasculature. The well-conserved role of PDGF-B signaling through its cognate PDGFR-β receptor in brain pericyte development was documented between mice [176–179] and zebrafish [174, 180]. CD146 is indicated to function as a co-receptor of PDGFR-β to mediate pericyte recruitment to cerebrovascular ECs and promote BBB maturation [181]. Notch3 expression in pericytes was shown to have a conserved role in maintaining BBB integrity and mural cell coverage of brain vasculature between mice [182] and zebrafish [183]. A recent study identified lactate, a metabolite produced through glucose metabolism in brain vECs, as a crucial energy source for pericytes in maintaining BBB function [119]. Emerging scRNA-seq data have identified potential new specific markers for brain pericytes in humans [81, 184, 185], mice [80, 186], and zebrafish [187], accelerating the study on brain pericyte heterogeneity in structure and function.

*Astrocytes* Astrocytes are another important cellular constitute of the BBB in mammals. Generation of astrocytes in the mammalian brain occurs postnatally after the functional BBB is established [158, 164], eliminating the possibility of an astrocyte role in endothelial acquisition of BBB properties during embryonic development. However, after passing through postnatal developmental stages, astrocytes can be critical for BBB maintenance and repair. A recent study indicated that mature astrocyte ablation from the mouse brain using the inducible astrocyte glutamate transporter *Glast-CreERT* line led to increased leakage of fluorescently labeled small molecule Cadaverine (< 1 kDa) into the brain parenchyma, suggesting BBB dysfunction following brain astrocyte ablation [188]. However, several studies using similar astrocyte-targeted genetic ablation systems with larger molecular tracers in the spinal cord did not report significantly increased BBB permeability [189, 190].

In the developing mouse brain, Reelin-induced activation of endothelial Dab1 plays an instructive role in directing astrocyte end-feet attachment to cerebral blood vessels for functional BBB development, as genetic inactivation of this signaling axis led to insufficient astrocyte end-feet coverage of these vessels, resulting in defective barrier properties [191]. This Reelin-endothelial Dab signaling is not critical for pericyte coverage and maintenance of the BBB [191], suggesting its developmental role in the assembly of the NVU. In adulthood, astrocyte-specific deletion of *Netrin-1* or *Neogenin* in mice resulted in increased BBB leakage and reduced pericyte vascular coverage [192], indicating a crucial role for astrocyte-derived Netrin-1 signaling in maintaining BBB and NVU integrity. Similarly, genetic inhibition of Wnt secretions from astrocytes led to impaired astrocyte end-feet morphology, reduced pericyte coverage, and increased levels of BBB permeability and endothelial transcytosis [193]. Thus, astrocyte-derived Wnts maintain BBB and NVU integrity after developmental stages by sustaining adequate levels of Wnt/β-catenin activity in brain vECs and astrocytes. Future studies will be needed to identify the specific Wnt ligand(s) secreted from astrocytes and their underlying cellular mechanisms responsible for maintaining the NVU integrity.

## Development and maintenance of endothelial fenestrations and permeable properties

VEGF signaling has been demonstrated as a key regulator of endothelial fenestrations in both in vitro [194] and in vivo [195]. VEGF-A signaling downregulates the expression of the tight junction proteins Claudin-5 and Occludin at the mRNA and protein levels [196], while it upregulates the mRNA and protein expression of PLVAP [197]. Across the CVOs, higher levels of *Vegf-A* mRNA expression were observed than in adjacent brain regions in adult mice [198]. Distinct cell types have been shown to regulate endothelial fenestrations across the CPs and CVOs.

In the ME, specialized ependymal cells, tanycytes, display increased Vegf-A expression in response to fasting, which results in increased levels of endothelial PLVAP expression, density, and fenestrations [199]. Recent work indicated that Melanin-concentrating hormone-expressing neurons, which extend axonal projections to the ME in close proximity to fenestrated capillaries, directly controls endothelial density and fenestrations via activity-dependent Vegf-A release from their axon terminals [200]. These results are consistent with a previous study that reported Vegf signaling-dependent continuous endothelial proliferation and angiogenesis in the ME of adult mice [201].

In the NH, Vegf-A signaling derived from pituicytes, glial cells of the posterior pituitary, controls continuous endothelial proliferation and angiogenesis in adult mice [202]. In the developing zebrafish NH, pituicyte-derived Vegf and TGF-β signaling induces *plvap* expression and high vascular permeability in its fenestrated capillaries [71]. In the same system, defective fenestrated vasculature forms in the pituitary in the absence of both the hypothalamic–hypophyseal axon tract and signaling evoked by the neuropeptide oxytocin secreted from its axon terminals [203].

In the CPs, several studies suggest a role for Vegf-A and TGF-β signaling derived from the ependymal epithelium in maintaining the integrity and fenestrations of capillaries in adult mice [195, 204]. Our recent study identified a unique combination of Vegf ligands required for driving fenestrated vascular development in the zebrafish hindbrain CP, while these ligands have little impact on the formation of neighboring BBB brain vasculature [73].

In the AP, OVLT, and SFO, Vegf-A expression in neurons and/or astrocytes is indicated to sustain continuous endothelial proliferation and vascular permeability of fenestrated capillaries in adult mice [198]. Additionally, in the AP, Wnt Inhibitory Factor-1 knockout mice displayed an elevated level of the GLUT1 BBB marker [29], suggesting that locally expressed endogenous inhibitors of Wnt/β-catenin signaling suppress vEC’s β-catenin activities to prevent BBB formation in the fenestrated vascular beds of the brain. Melatonin, a hormone secreted by the PG, has been implied in the regulation of angiogenesis [205], especially inhibition of multiple tumor angiogenesis; however, its role in fenestrated capillary development or maintenance in the PG remains unclear.

## Perivascular cell diversity and their emerging roles in controlling endothelial properties

Recent scRNA-seq data have revealed transcriptionally diverse subtypes of brain perivascular cells, including pericytes [80, 81, 184], fibroblasts [80, 82, 206, 207], smooth muscle cells [80, 186, 208], as well as macrophages and microglia [209–212]. An increasing number of scRNA-seq datasets that contain brain perivascular cell populations have been reported under physiological and pathological conditions across species, developmental stages, and brain regions. However, except pericytes, the functional roles of each perivascular cell type in regulating brain vEC properties remain largely unknown.

A recent study showed that perivascular fibroblasts lie adjacent to smooth muscle cells in arterioles and large-diameter venules, but are absent in capillaries, in the adult mouse brain [213]. scRNA-seq results of embryonic mouse brain meninges identified transcriptionally distinct fibroblast subtypes derived from different embryonic origins in brain meningeal compartments [206]. Our recent work identified mesoderm-derived meningeal fibroblasts as important sources of Vegf ligands critical for directing fenestrated vessel formation in the zebrafish CP [73]. Future studies will be needed to uncover how fibroblast subtypes, including perivascular fibroblasts, contribute to the establishment of brain vascularization and the endothelial acquisition of unique properties across the brain.

Similarly, recent scRNA-seq data have indicated the unique transcriptional signatures of perivascular macrophages, which are distinguishable from those of microglia, in the mouse cortex [212]. This and other emerging scRNA-seq results have provided valuable information to identify and establish reliable cell type-specific markers for perivascular cells. Given the unique distributions of perivascular cell types along vEC types and across brain regions [175, 213, 214], future studies on dissecting the functional roles of each cell population and subtype in regulating vEC properties across the brain will expand our understanding of dynamic and heterogeneous brain vEC properties.

## Current and emerging models for the fate determination of brain endothelial cells

**Fig. 3:**
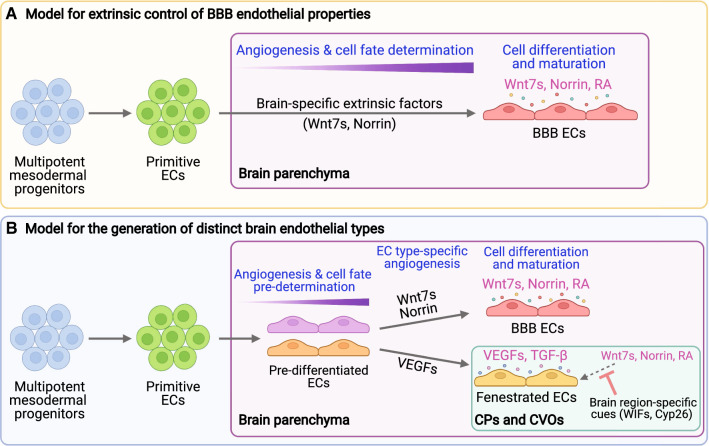
Current and emerging models for the fate determination of brain endothelial cells. **A** In a prevailing model, brain-specific signaling induced by extrinsic factors, such as Wnt7s and Norrin, induces the differentiation of primitive endothelial cells into a BBB EC type as they migrate to target brain regions. **B** In an emerging model, primitive endothelial cells first undergo pre-differentiation, which allows them to migrate to target brain regions in response to specific sets of angiogenic cues via EC type-specific angiogenesis. In the CPs and CVOs where fenestrated ECs are generated and maintained, BBB inducing extrinsic signals, such as Wnt7s, Norrin, and RA, are suppressed by Wnt inhibitory factors and/or RA degrading enzymes, Cyp26. *BBB* blood–brain barrier, *CPs* choroid plexuses, *CVOs* circumventricular organs, *Cyp26* cytochrome P450 family 26 enzymes, *ECs* endothelial cells, *RA* retinoic acid, *TGF-β* transforming growth factor beta, *VEGFs* vascular endothelial growth factors, *WIFs* Wnt inhibitory factors

In light of classical studies that conducted chick-quail transplantation experiments, neural environmental signals or extrinsic factors are thought to be the key determinants of brain vEC barrier properties [132, 133]. In support of this finding, additional studies have established that canonical Wnt/β-catenin signaling is necessary [114, 135] and sufficient [29, 30] to induce and maintain BBB properties in brain vECs (Fig. 3A). However, it remains unknown whether neural environmental signals are necessary and sufficient to induce a fenestrated endothelial cell fate and if so, what the molecular determinants of this cell fate are. This incomplete knowledge has limited our current understanding of brain vEC fate specification across the brain, leaving open the question of whether BBB and fenestrated vEC properties in the brain are determined entirely by extrinsic signals.

There still remains the possibility that the distinct fates of brain vECs derive from different embryonic origins. Our recent results indicate that during brain vascularization in zebrafish, adjacent vECs exhibit individually distinct responses to local Vegf ligands, resulting in different fates [73]. These findings suggest that brain vECs undergo fate pre-determination prior to angiogenesis and that these intrinsic properties facilitate migration of vECs to their destinations in response to environmental angiogenic factors via “EC type-specific angiogenesis” (Fig. 3B). Thus, it is possible that a combination of intrinsically programed pre-determination and dynamic spatiotemporal presentation of local morphogenetic cues direct fate decisions and heterogeneous network formation of brain vECs. This model is in line with a recent study that demonstrated lineage history as a critical determinant of EC specialization [95]. The authors reported that the lymphatic lineage derived from the paraxial mesoderm contributes to lymphatic endothelium in multiple organs, but in an organ-restricted manner [95], indicating that EC fate is imprinted before the established endothelial genetic specification process is initiated. Identifying the key developmental determinants of fenestrated brain vEC identities will accelerate our understanding of the mechanisms underlying the generation of brain vEC heterogeneity.

## Plasticity of brain endothelial fates and states under physiological and experimental conditions

Endothelial fates and states are plastic during development and in adulthood [215–217], resulting in heterogeneous permeability of this cell type. Neuronal activity has been proposed as a crucial modulator of influx [218] and efflux [219] transport activities in BBB vECs, thereby controlling BBB permeability dynamics [220]. In addition, efflux transporter function in BBB vECs undergoes circadian regulation [219, 221, 222], inducing circadian changes in BBB permeability throughout the day. Since the production of certain molecules and metal ions such as hormones, neuropeptides, cytokines, and magnesium has been reported to undergo circadian oscillations [223], the rhythmic expression of these molecules and ions may mediate circadian changes in BBB permeability. Sleep loss also causes BBB permeability changes through down-regulation of tight-junction protein expression [224] and levels of endocytosis [225] in brain vECs. Other physiological factors that modulate BBB tightness include pregnancy, nutrition, body temperature, physical activity, gut microbiota, and psychological stress [226].

In contrast to our increasing knowledge of BBB permeability dynamics and their modulators, much less is known about physiological states that modulate fenestrated vEC permeability in the brain. One study showed that fasting, or glucose deprivation, increases PLVAP expression and endothelial fenestrations in fenestrated capillaries of the murine ME [199]. The activity of hypothalamic neurons involved in food intake, locomotor activity, and sleep also controls fenestrated endothelial density and permeability in the ME [200], suggesting that these physiological factors modulate vEC permeability states in this brain region. Seasonal changes in the length of daylight (i.e., photoperiod) lead to the oscillatory pattern of melatonin secretion from the PG, which is indicated to affect fenestrated vascular density in the sheep ME [227].

Recent studies in mice demonstrated that fenestrated brain vECs respond differentially to forced activation of β-catenin across the CVOs and CPs in terms of their phenotypic conversions into a BBB state [29, 30], suggesting that heterogeneity in the phenotypic plasticity of vECs exists across fenestrated vascular beds of the brain. Changes in fenestrated brain vEC permeability can influence fluid balance, waste clearance, immune surveillance, and the efficiency of hormonal secretion and reception, through this vessel type. However, fenestrated vascular permeability dynamics have been understudied. Future studies on structural and functional changes in fenestrated brain vasculature in response to physiological and pathological stimuli will advance our knowledge of the plasticity of this vEC type.

## Pathological and age-induced changes in brain endothelial properties and heterogeneity

Significant structural and functional changes of brain capillaries have been reported with aging and in disease states [2, 228–230]. For example, the brain of elderly humans [231] and of aged mice [22] displays capillary wall thinning and declines in capillary density, blood flow, vascular perfusion, angiogenic potentials, and barrier properties [16–18]. Decreased pericyte coverage of brain vasculature and diminished *Mfsd2a* expression were also observed with aging [22]. Moreover, pericyte degeneration is associated with BBB disruptions in patients with neurological diseases such as AD and amyotrophic lateral sclerosis (ALS) [232–235]. Since brain pericytes regulate blood flow via capillary constriction [236, 237], reduced cerebral blood flow as a consequence of pericyte degeneration is another hallmark associated with aging and age-related neurodegenerative diseases [238]. In support of these observations, forced pericyte ablation with diphtheria toxin using an inducible pericyte-specific Cre line in mice led to rapid BBB breakdown and neurodegeneration [239].

While brain pericyte deficiency clearly promotes BBB disruptions, viable pericytes can also produce numerous proinflammatory mediators that are detrimental to BBB function [240]. In aging human and rodent brains, increased production of the pleiotropic cytokine TGF-β was reported [241], which can upregulate a number of inflammatory genes in pericytes in vitro [242]. Conditional loss of the transcription factor RBPJ in pericytes of mouse brains triggers excessive production of TGF-β3 that leads to overexpression of inflammation-related genes, suggesting a potential role for RBPJ in regulating a proinflammatory genetic program in pericytes [243]. Notably, pharmacological inhibition of TGF-β signaling restored cognitive impairments in aged mice [241] and ameliorated autoimmune encephalomyelitis in a mouse model [244], indicating that TGF-β signaling inhibition counteracts detrimental consequences of neuroinflammation.

There is a growing body of transcriptomic datasets that offer enriched information on gene expression changes of brain vECs over the course of development, and also in response to disease conditions. A recently established transcriptome database of isolated mouse brain vECs under healthy and various neurological disease states identified a common, core pathway leading to BBB dysfunction regardless of BBB disruption triggers [79]. This common BBB dysfunction module among diseases includes upregulation of genes that are enriched in vECs of peripheral organs under normal physiological conditions, indicating a shift of the BBB vEC identity toward peripheral non-BBB cell states [79]. Moreover, single-cell atlases of human brain vasculature from individuals with AD [245], Huntington’s disease [185], or arteriovenous malformations [81] have provided valuable resources to understand vascular cell-type-specific perturbations of gene expression under these disease states compared to healthy individuals.

In contrast to BBB dysfunction linked to aging and neurological disorders, very little is known about structural and functional alterations of fenestrated brain capillaries under these conditions. Given that defective brain–blood communications via fenestrated vasculature can lead to impaired metabolic sensing and hormonal release into the bloodstream, it is likely that structural and functional changes in this vEC type abrogate neuroendocrine control and action. Indeed, alterations of neuroendocrine function with aging have been documented [246, 247], which include imbalanced hormone production [248] and reduced signaling reception and sensitivity for secreted hormones [249]. Considering age-related VEGF signaling declines [21] and the crucial role of this signaling in maintaining endothelial fenestrations in the CPs and CVOs [71, 195, 204], it is likely that endothelial fenestration numbers, sizes, and permeability in these brain regions decline with aging and that these age-related changes affect neuroendocrine function. Future investigations into age-related changes in the number, size, and/or permeability of endothelial fenestrations across the CPs and CVOs will help understand the malfunction and disease associated with these brain regions.

## Development of potential therapeutics targeting unique brain endothelial properties

Pharmacological inhibition of the PDGF-C/PDGFR-α signaling axis has been pursued as a powerful approach to restore BBB dysfunction in neurological disorders [27]. Previous studies showed that the intracerebroventricular injection of either PDGF-C or tissue plasminogen activator was sufficient to increase BBB permeability and induce BBB dysfunction [250, 251]. Conversely, inhibition of the PDGF-CC signaling by neutralizing antibodies or inhibition of PDGFR-α with imatinib reduced BBB dysfunction in mouse models of ischemic stroke [250, 252], MS [253, 254], seizure [255], and traumatic brain injury [256]. Thus, preserving BBB integrity by targeting this signaling axis is a promising therapeutic approach for a wide range of neurodegenerative and neuroinflammatory diseases associated with BBB breakdown.

Strong neuroprotective effects of activated protein C (APC) on both acute brain injury and chronic neurodegenerative conditions make it another potential therapeutic target [28]. Intravenous injections of APC were shown to exert beneficial therapeutic effects in mouse models of ischemic stroke [257], MS [258], ALS [259], and AD [260]. It has been proposed that APC functions by eliciting anti-inflammatory effects and neuroprotective actions within the NVU to prevent BBB breakdown [28].

Another therapeutic strategy is the repurposing of a key developmental BBB induction signal as a BBB protective agent for neurological pathology [26]. This successful translation of developmental biology knowledge into potential therapies opens a new avenue for developing therapeutics for a variety of neurological and cerebrovascular diseases, as well as for age-related vascular declines in the brain. By conducting a screen of the numerous Wnt7a variants generated via single-residue substitutions, the authors identified genetically engineered Wnt7a ligands that can preferentially activate the Gpr124/Reck co-receptor complex while dramatically reducing activation of its other cognate Frizzled receptors [26, 144]. This study further demonstrated that these Gpr124/Reck-specific activators acted as BBB repair agents and mitigated the progression of neurological disease in mouse models, including glioblastoma and stroke. This finding is consistent with a separate study where endothelial-specific β-catenin stabilization improves BBB function in conditional Gpr124-deficient mice under pathological neurological conditions [261].

Emerging therapeutic targets include the Netrin1-Unc5B signaling axis that is indicated to act upstream of canonical β-catenin pathways in the brain [148]. Current evidence shows that *Netrin-1* global knockouts [262] or endothelial-specific deletion of the *Unc5B* receptor in mice [148] led to the down-regulation of BBB markers in vECs, while upregulating their PLVAP expression [148]. In contrast, Netrin-1 treatments can enhance BBB marker expression both in vitro and in vivo [148, 192, 262] and reduce BBB leakage in mouse models of MS [262] and BBB breakdown [192], suggesting BBB protective effects of Netrin-1-Unc5B signaling.

**Fig. 4:**
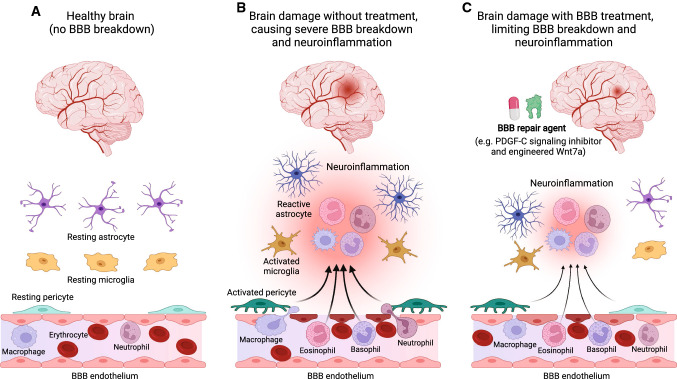
Expected therapeutic effects of a BBB repair agent(s) on brain disease/injury associated with BBB dysfunction. (**A**) In a healthy brain, no neuroinflammation and pathological BBB breakdown are detected. (**B**) In a diseased/injured brain without any treatment, severe neuroinflammation and BBB breakdown cause serious brain damage. Brain disease/injury elicits a series of neuroimmune responses via activation of microglia, astrocytes, and pericytes into proinflammatory states [240] that lead to their release of inflammatory mediators involved in BBB disruptions. This permits blood-circulating immune cell infiltration into the damaged brain region. These neuroinflammatory events induce neurological damage. (**C**) In a diseased/injured brain subjected to treatment with a BBB repair agent, mitigated neuroinflammation and BBB breakdown cause only limited brain damage. As compared to the scenario described in (**B**), timely administration of a BBB repair agent, such as PDGF-C signaling inhibitor or engineered Wnt7a ligand, can ameliorate the damaging effects of neuroinflammation by limiting BBB disruptions. *BBB* blood–brain barrier, *PDGF-C* platelet-derived growth factor C

The four strategies discussed above all help mitigate BBB dysfunction, thereby preventing the progression and worsening of neuroinflammation and BBB breakdown that are the hallmarks of a broad range of neurological diseases (Fig. 4). Future studies may seek to test whether treatment approaches targeting different signaling pathways could have additive or synergistic therapeutic effects on these diseases. Furthermore, our increasing knowledge of brain vEC’s transcriptomic changes under a variety of disease conditions and their shared BBB dysfunction module [79, 245] can help identify potential new therapeutic options for BBB repairs that may stand alone or boost the effects of the existing approaches.

In many pathological and developmental conditions where endothelial BBB integrity and identities are disrupted, upregulation of PLVAP expression in vECs has been reported. For example, PLVAP upregulation is detected in microvasculature associated with brain tumors and ischemia [263–265] where neuroinflammation and BBB breakdown occur, or in vasculature where reduced levels of endothelial β-catenin signaling are observed [114, 138, 140, 148]. These observations are consistent with the current model that active β-catenin signaling suppresses PLVAP expression in brain vECs to establish the BBB [29, 30, 114]. Since anti-PLVAP therapy has been explored as a potential anti-angiogenesis and anti-edema therapeutic option for cerebral edema caused by ischemic stroke and brain tumors [110, 111], this option may be pursued to limit vascular leakage and BBB breakdown in conjunction with the other BBB repair strategies. However, further investigations will be needed to define the cellular and molecular mechanisms underlying PLVAP upregulation and its contributions to vascular leakage and BBB breakdown during aging and neuroinflammation.

Beyond BBB-targeted therapies, efficient drug delivery across the BBB has remained a significant challenge in treating or preventing the progression of neurological deficits. Efforts have been made to exploit endogenous receptor-mediated transcytosis pathways to enhance uptake of large molecules and therapeutics in the brain [266, 267]. Transferrin/transferrin receptor (TFRC) pathway is one of the major transport pathways specific to the brain and BBB [268–270]. Since TFRC is highly expressed in BBB vECs [268, 269] and brain tumors such as glioblastomas [271–273], nanoparticles targeted with TFRC-binding peptides have been explored as diagnostic probes for imaging brain tumors [274, 275] and for boosting brain uptake of therapeutic antibodies [276–278]. Another strategy for promoting drug delivery across the BBB includes the use of the monoclonal antibodies that can block specific Netrin-1-Unc5B interactions or induce Unc5B internalization [148]. These Unc5B blocking agents were suggested to enhance size-selective BBB permeability within a limited time window [148], indicating their potential application to transient BBB opening for therapeutic uptakes in the brain.

In contrast to the promising BBB repair approaches, current molecular targets for repairing fenestrated brain vECs are substantially limited. PLVAP is a well-established marker for fenestrated brain vECs and currently known to be the only structural components of fenestral and stomatal diaphragms [109–111]. In conditions where high vascular permeability in fenestrated brain vECs declines due to reduced numbers of endothelial fenestrations with aging and/or disease, restorations of fenestrations can be possibly achieved by targeting PLVAP itself, or VEGF and/or TGF-β signaling, which are indicated to be required for induction of PLVAP expression during development [71] and maintenance of endothelial fenestrations in adults [195, 204]. However, systemic upregulation of PLVAP expression can be problematic because this may result in a significant increase in overall brain vascular permeability or unwanted angiogenesis due to the potential role of PLVAP in developmental and pathological angiogenesis [72, 263].

Recent bulk and single-cell transcriptomes have indicated many other genes that are highly expressed in fenestrated brain vECs [10, 29, 279], including *Plpp1*, *Plpp3*, *Igfbp3*, *Cd24a*, and *Ldb2*. Future studies on these and other candidate genes in the development and/or maintenance of fenestrated brain vEC will shed new light on potential new targets for repairing this cell type.

## Future perspectives

Brain barriers act as the important boundaries that separate the brain parenchyma from the periphery across species. Multiple blood vEC types present in the brain play a central role in establishing and maintaining some of these barriers, thereby ensuring an optimal brain microenvironment. In addition to the widely recognized BBB properties that a majority of brain vECs exhibit, fenestrated vECs possess unique barrier properties across the CVOs and CPs. Numerous factors can affect the states of brain vECs globally or locally, thereby generating the heterogeneity and highly dynamic changes in their vascular permeability. The evolution of technology has enabled us to explore more diverse scientific approaches and research directions than in the past. Here, we discuss some emerging approaches untouched in earlier sections that will help address unsolved questions in the field.

Recently, there have been significant advances in in vitro model systems. These include the vascularization models of human brain organoids-on-a-chip [280, 281], self-assembling multicellular BBB spheroids [282, 283], and improved endothelial BBB differentiation protocols using human pluripotent, or induced pluripotent, stem cells [284, 285]. Human organoid and cell reprogramming technologies have been rapidly expanding to mimic human brain development and disease modeling with the construction of a functional vasculature. These in vitro models will become powerful tools for studying human-specific vascular traits absent in animal models, drug delivery across the BBB, and as a drug-screening platform for ameliorating BBB dysfunction in neurological diseases.

Emerging large-scale biological resources from multi-omics data have increased the demand for an efficient in vivo platform to screen numerous candidate genes emerging from big data analytics. Given the high conservation of the molecular mechanisms underlying brain vascularization across vertebrates, one effective approach includes the use of the zebrafish system, which provides technical advantages such as rapid *ex utero* development and facile 3D visualization of brain vasculature. Recently, our group, along with others, reported highly efficient and scalable CRISPR/Cas9-based mutagenesis protocols to generate F0 zebrafish knockouts [73, 286, 287]. These technical advantages will make the zebrafish a valuable model to perform phenotype-based F0 screens of many candidate genes individually, or in combinations, in vivo.

The identification of brain regional differences in BBB or fenestrated vEC barrier properties has indicated brain area-specific regulations of brain vEC characteristics mediated by their surrounding cellular compositions (e.g., perivascular, glial, and/or neuronal types). Cutting-edge technologies, such as spatial transcriptomics [288], combined with an in situ sequencing method [289] will enable the visualization and analysis of many transcripts with single-cell resolution [290–292]. These approaches will compensate for the lack of positional information on emerging single-cell and bulk transcriptome data of brain vECs and perivascular cell types in health and disease, allowing for spatial mapping of gene expression profiles in situ.

The discovery of novel regulators of BBB permeability in recent years has illuminated potential new targets for delivering therapeutics across the BBB. Endothelial transcytosis pathways have been a major target of non-invasive brain delivery approaches [293]. Several recently identified molecules (Mfsd2a, Vitronectin, and Integrin α5) critical for suppression of transcytosis in BBB vECs [158–161, 163] have become attractive targets for non-invasive drug transport into the brain parenchyma. For example, drugs that inhibit MFSD2A activity may release the suppression of transcytosis in BBB vECs, thereby increasing endothelial molecular transport via transcytosis into the brain parenchyma. Recent advances in structural biology and protein design methods [294–298] will facilitate in silico structure-based molecular designs that can enhance the efficiency of drug delivery across the BBB.

The approaches listed here represent only a few examples of emerging directions in the field that have evolved with technological advances. Cutting-edge multi-omics platforms have offered the opportunity to simultaneously profile RNA and DNA/protein at the single-cell level and with spatial resolution, becoming increasingly powerful tools to study the heterogeneous identities and states of brain vascular cell types in health and disease. Future investigations into the complex physiology and pathology of brain–blood interfaces using multifaceted approaches will reveal new therapeutic horizons for treatments of cerebrovascular and neurological diseases.

## Data Availability

Not applicable.

## Abbreviations

AD: Alzheimer’s disease
ALPM: Anterior lateral plate mesoderm
ALS: Amyotrophic lateral sclerosis
AP: Area postrema
APC: Activated protein C
BBB: Blood–brain barrier
CNS: Central nervous system
CPs: Choroid plexuses
CVOs: Circumventricular organs
Cyp26: Cytochrome P450 family 26 enzymes
EMPs: Erythro-myeloid progenitors
ME: Median eminence
MS: Multiple sclerosis
NH: Neurohypophysis
NVU: Neurovascular unit
OVLT: Organum vasculosum of the lamina terminalis
PDGF-B: Platelet-derived growth factor B
PDGF-C: Platelet-derived growth factor C
PG: Pineal gland
PLVAP: Plasmalemma vesicle-associated protein
RA: Retinoic acid
SCO: Subcommissural organ
SFO: Subfornical organ
TGF-β: Transforming growth factor beta
TFRC: Transferrin receptor
vECs: Vascular endothelial cells
VEGFs: Vascular endothelial growth factors
